# Complete genome sequence of a multi-drug-resistant *Mycobacterium avium* subspecies *hominissuis* isolated from a patient with lung infection

**DOI:** 10.1128/mra.00014-26

**Published:** 2026-03-05

**Authors:** Franck Biet, Claire Hamela, Francoise Roquet-Baneres, Sandrine Melo, Thierry Cochard, Sonia Lameiras, Sylvain Baulande, Helene Chiapello, Valentin Loux, Laurent Kremer

**Affiliations:** 1 INRAE, UMR ISP, Université de Tours 56586 , Nouzilly, France; 2 Centre National de la Recherche Scientifique UMR9004, Institut de Recherche en Infectiologie de Montpellier (IRIM), Université de Montpellier, Montpellier, France; 3 Institut Curie, PSL University, ICGex Next-Generation Sequencing Platform 56586 , Paris, France; 4 Institut Curie, PSL University, Single Cell Initiative 74288 , Paris, France; 5 Université Paris-Saclay, INRAE, MaIAGE 55216, Jouy-en-Josas, France; 6 Université Paris-Saclay, INRAE, BioinfOmics, MIGALE Bioinformatics Facility, Jouy-en-Josas, France; 7 INSERM, IRIM, Montpellier, France; Wellesley College, Wellesley, Massachusetts, USA

**Keywords:** *Mycobacterium avium* subspecies *hominissuis*, multi-drug-resistance, complete genome

## Abstract

The complete genome sequence of the strain GD386 of *Mycobacterium avium* subspecies *hominissuis* isolated from a patient with lung infection in France was determined. The genome was sequenced using the PacBio technology, yielding a genome size of 5,562,671 nucleotides with no identified plasmids.

## ANNOUNCEMENT

*Mycobacterium avium* subspecies *hominissuis* is a slow-growing nontuberculous mycobacterium of high clinical significance in humans (1, 2). It is responsible for pulmonary and soft tissue infections, lymphadenitis, as well as disseminated diseases (2). The near absence of effective multidrug regimens renders treatment of *M. avium* subspecies *hominissuis* infections very challenging, urging for new effective drug regimens (3). Pathogenicity as well as responsiveness to chemotherapy largely depend on bacterial characteristics as well as host and environmental factors (4).

**Fig 1:**
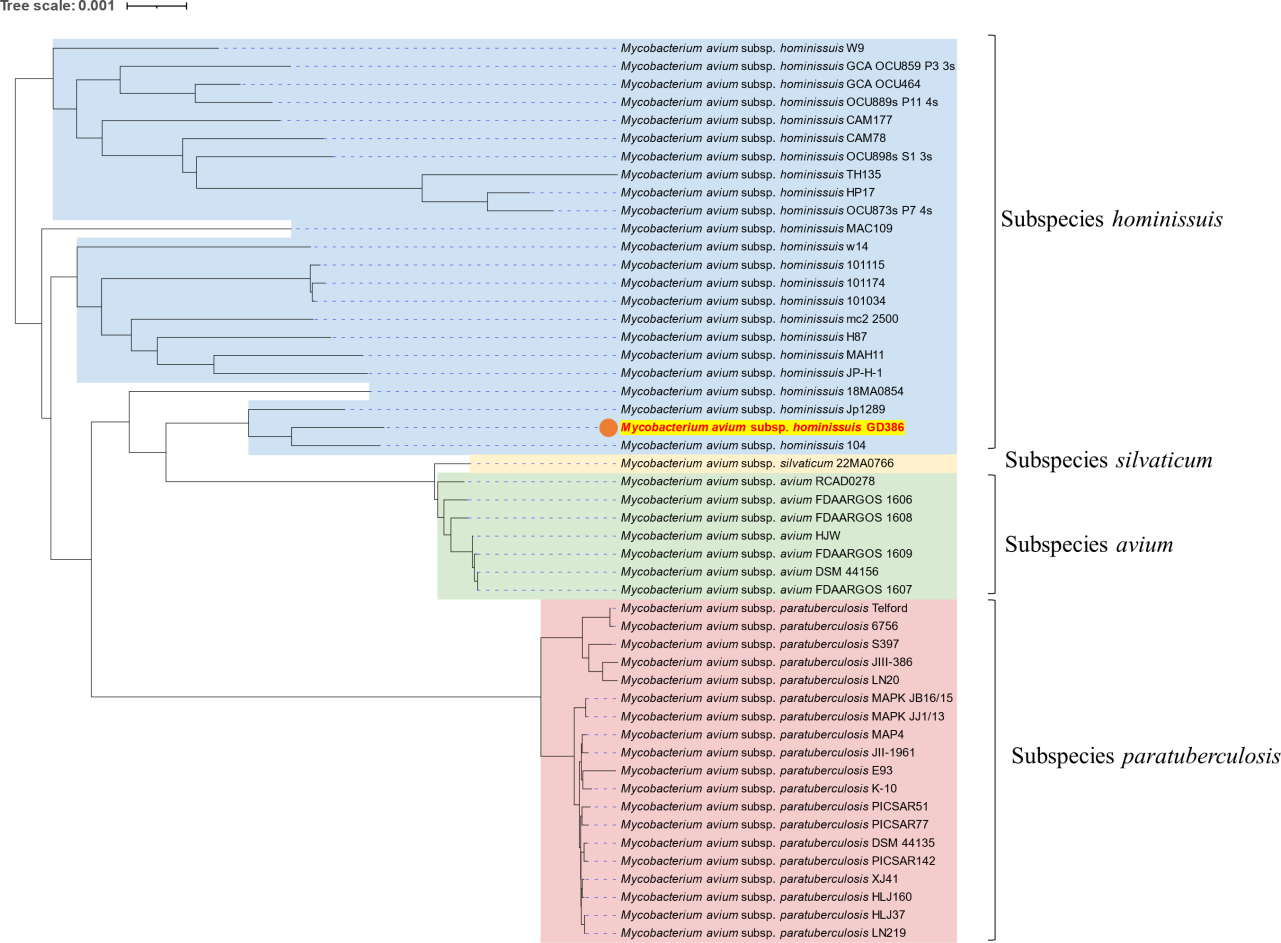
Clustering analysis of *Mycobacterium avium* subspecies *hominissuis* GD386 strain. The figure shows the position of the *Mycobacterium avium* subspecies *hominissuis* GD386 within the phylogeny of the *Mycobacterium avium* species. The phylogenetic tree was inferred with 49 complete genome sequences extracted from GenBank in November 2025 and the genome of the *Mycobacterium avium* subspecies *hominissuis* GD386 and uploaded to the Type Strain Genome Server (TYGS), available at https://tygs.dsmz.de. The phylogenetic tree was displayed and annotated using the online tool Tree of Life (iTOL) v6 (6). The genome of the GD386 strain of *Mycobacterium avium* subspecies *hominissuis* is indicated by a red dot and text.

Here, we report the complete genome sequence of *Mycobacterium avium* subspecies *hominissuis* GD386 (Fig. 1), isolated from a 68-year-old female patient with lung infection who was under therapeutic failure after 18 months of treatment with clarithromycin, rifampicin, and ethambutol, followed by an additional 6-month treatment with these three antibiotics combined with nebulized liposomal amikacin (5). Treatments were stopped due to severe side effects. This strain was isolated from a sputum sample in France in 2018 and grown on Middlebrook 7H11 supplemented with oleic acid-albumin-dextrose-catalase enrichment (Becton Dickinson) for 3 weeks at 37°C. Research conducted with this strain was carried out in accordance with our institution’s guidelines and the Declaration of Helsinki.

Bacteria were harvested by centrifugation at 4,000 × *g* for 15 min. The pellet was resuspended in 1 mL PBS with 20 mg lysozyme (Sigma 62,971) and incubated at 37°C overnight with shaking. Genomic DNA was extracted using the kit MagAttract HMW DNA (Qiagen) with 1% SDS according to the manufacturer’s instructions for gram-negative bacteria. Extracted DNA was quantified using a Qubit4 fluorometer (Thermo Fisher Scientific).

For Pacific Biosciences sequencing, DNA was sent to the ICGex NGS platform (Institut Curie, France). SMRTbell libraries were prepared from 5 μg of sheared DNA, using the SMRTbell Prep Kit 3.0, following PacBio’s instructions. DNA was sheared using the Megaruptor2 (Diagenode) with a setting at 30 kb. Then, we applied a purification step with SMRTbell Cleanup beads and assessed the fragmentation profile on a Femto Pulse. The fragments obtained showed a mean size of 15 kb. Library sequencing was performed using the SMRTcell of Sequel II System with sequencing plate v2.0.0, producing 168,506 reads representing 1.7 Mbp. Raw reads were filtered and quality-controlled using fastplong 0.2.2 (7) (default parameters). After filtering, we obtained 137,608 high-quality reads, N50 of 12,573 nct, representing 1.2 Mbp of sequence. *De novo* assembly was performed with Autocycler 0.5.0 (8) (default parameters, estimated genome size of 4.8 Mbp) using Flye 2.9.6-b1802 (9), Raven 1.8.3 (10), and miniasm 0.3-r179 (11). The genome was circularized using Dnaapler 1.2.0 (12). Quality control of assembly was performed using Quast 5.3.0 (13), Checkm2 1.1.0 (14), and GTDB-Tk 2.4.0, database release 220. The genome was indicated as “fully resolved” and “circular” by Autocycler, a completeness of 100% by CheckM2, and correctly assigned taxonomically by GTDB-Tk. Genome annotation was completed using the NCBI Prokaryotic Genome Annotation Pipeline (PGAP version 6.5) (15–17) and includes a total of 5,290 genes and 49 tRNAs.

## Data Availability

This Whole Genome Shotgun project was deposited at DDBJ/ENA/GenBank under accession number JBSLYY000000000. The version described in this paper is version JBSLYY010000000. The original sequence reads were deposited in the Sequence Read Archive under accession number SRR36220032.
